# Egg antigen p40 of *Schistosoma japonicum* promotes senescence in activated hepatic stellate cells by activation of the STAT3/p53/p21 pathway

**DOI:** 10.1038/cddis.2016.228

**Published:** 2016-07-28

**Authors:** Jinling Chen, Tianhua Xu, Dandan Zhu, Jianxin Wang, Caiqun Huang, Lei Lyu, Bin Hu, Wei Sun, Yinong Duan

**Affiliations:** 1 Department of Pathogen Biology, School of Medicine, Nantong University, Nantong, Jiangsu 226001, People's Republic of China; 2 Laboratory Medicine Center, Affiliated Hospital of Nantong University, Nantong, Jiangsu 226001, People's Republic of China

## Abstract

Liver fibrosis is a serious disease that is characterized by the excess deposition of extracellular matrix (ECM) components. Activated hepatic stellate cells (HSCs) are a major source of ECM and serve as a key regulator in liver fibrogenesis. Inactivation of HSCs is essential for liver fibrotic regression. The present study explores the underlying mechanisms of *Schistosoma japonicum* egg antigen p40 (Sjp40) promoting senescence in HSCs and antifibrosis. For the first time we report that Sjp40 inhibits the activation and proliferation of an immortalized human HSC line (LX-2 cells) and promotes cellular senescence and cell cycle arrest. Sjp40 through action on the STAT3/p53/p21 pathway triggered cellular senescence, while knockdown of p53 or STAT3 partly restored cell senescence. In addition, Sjp40-induced cellular senescence caused LX-2 cells to be more sensitive to a human NK cell line (YT cells). Together these findings provide novel insights into the mechanism of antifibrosis and may have implications for the development of antifibrosis therapies.

Liver fibrosis is defined as the excess deposition of extracellular matrix (ECM) components, including fibronectin and collagen, that leads to cirrhosis, liver failure and portal hypertension in advanced hepatic fibrosis.^1, 2^ It is widely accepted that activated hepatic stellate cells (HSCs) are a major source of the ECM and play a central role in liver fibrogenesis. HSCs undergo a transformation from a quiescent cell to a myofibroblast that can produce a great deal of ECM and secrete large amounts of pro-inflammatory and pro-fibrogenic cytokines.^3, 4^ Therefore, the inhibition of HSC activation and the removal of activated HSCs have been effective strategies used to combat hepatic fibrosis.^5, 6^ In recent years, the role of senescence in activated HSCs has been explored, and studies have found that HSCs that underwent cellular senescence resulted in liver fibrosis regression.^7^ These data suggest that the induction of senescence in activated HSCs may be a promising approach for treating hepatic fibrosis.

Schistosomiasis is a parasitic disease characterized by egg deposition, a granulomatous inflammatory reaction and subsequent hepatic fibrosis formation.^8, 9^ However, the antifibrotic effect of *Schistosoma* eggs and soluble egg antigens (SEA) on activated HSCs has been demonstrated in both *Schistosoma mansoni* eggs and *Schistosoma japonicum* eggs. These eggs could restrict the activation of HSCs during hepatic fibrogenesis.^10, 11^ Our previous research demonstrated that SEA from *S. japonicum* induced suppression of activated human HSC cell lines (LX-2) and primary mice HSCs through the TGF*β* and PPAR*γ* signaling pathways.^12^ SEA-treated LX-2 and primary HSCs exhibited cell cycle arrest, cell growth inhibition, and both caspase-^12^ and p53/DR5-dependent apoptosis.^13^

SEA is a complex mixture that is composed of a number of egg antigens. Some laboratories have isolated multiple antigens from SEAs, including Smp40 (*S. mansoni* egg antigen p40) and Sjp40 (*S. japonicum* egg antigen p40). Smp40 has been cloned, sequenced and shown to have high immunogenicity in humans.^14^ The Sjp40 antigen may be a promising target for prevention and control of the disease following its discovery as a marker for early schistosomiasis diagnosis.^15^ Sjp40 has also been observed to markedly increase IL-10 and significantly reduce IL-5 in Smp40-treated peripheral blood mononuclear cells from patients infected with *S. japonicum.*^16^ In addition, other studies have been carried out that support a role for IL-10 and IL-5 in hepatic fibrosis. Research has demonstrated that IL-10 could reverse hepatic fibrosis by attenuating the expression of matrix metalloproteinase and collagen.^17, 18^ More work also showcased the ability of IL-5 to promote the progression of hepatic fibrosis by regulating IL-13 activity.^19^ Together, these observations support a model in which Sjp40 might modulate liver fibrosis and exert an antifibrosis effect.

In previous research by our laboratory we expressed and purified Sjp40 and used this antigen to stimulate LX-2 cells *in vitro.*^20^ Our results confirmed that Sjp40 potently inhibited the activation of HSCs and combated liver fibrosis. We also demonstrated, for the first time, that Sjp40 could induce cellular senescence in LX-2 cells. In this work we set out to clarify the role of Sjp40-induced senescence in LX-2 cells and elucidate the underlying molecular mechanism.

## Results

### Sjp40 inhibits LX-2 cells activation in culture

**Figure 1:**
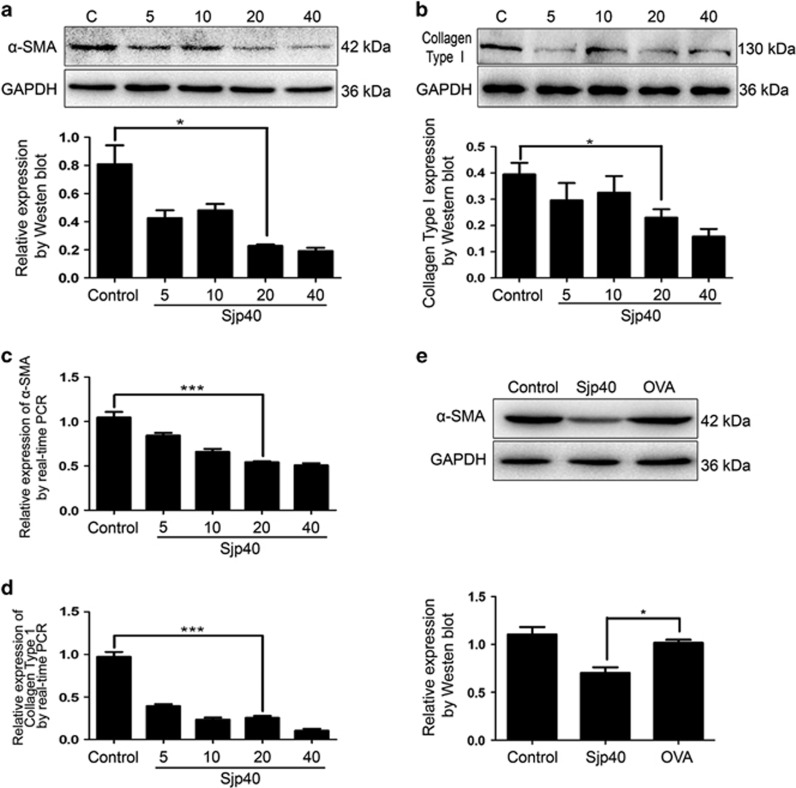
Sjp40 treatment for 48 h suppresses cell activation through the downregulation of *α*-SMA and collagen type I. (**a** and **b**) Protein expression of *α*-SMA and collagen type I in LX-2 cells treated with different concentrations of Sjp40 (5, 10, 20 and 40 *μ*g/ml) for 48 h were analyzed by western blot. (**c**) The expression of *α*-smooth muscle actin mRNA was inhibited by Sjp40 treatment in LX-2 cell. (**d**) The expression of collagen type I mRNA was inhibited by Sjp40 treatment in LX-2 cell. (**e**) Protein expression of *α*-smooth muscle actin in primary activated hepatic stellate cells was diminished by Sjp40 treatment. The graph also shows quantitative analysis of bands by Image J. All values were expressed as the mean±S.E.M. of three or four independent trials. *P<0.05 compared with the normal control group or OVA negative control group. ***P<0.001 compared to control group

It is widely accepted that the increased expression of collagen type I and *α*-SMA are markers of HSC activation.^2^ The recombinant Sjp40 protein was expressed and purified (Supplementary Figure S1). To explore the effect of Sjp40 on the activation of LX-2 cells we exposed LX-2 cells at different concentrations of Sjp40 (5, 10, 20 and 40 *μ*g/ml) for 48 h. The mRNA and protein expression of *α*-SMA and collagen type I in LX-2 cells markedly decreased in a dose-dependent manner following treatment with Sjp40 for 48 h (Figures 1a–d). Sjp40 also inhibited collagen type I in a dose-dependent manner compared with the control group. To further test the role of Sjp40 on primary HSCs *in vitro*, primary HSCs were isolated and activated by TGF*β*1 (5 ng/ml). We found that Sjp40 obviously inhibited *α*-SMA in primary HSCs (Figure 1d). All together, these findings indicate that Sjp40 inhibits HSC activation *in vitro.*

### Sjp40 induces cellular senescence in HSCs

**Figure 2:**
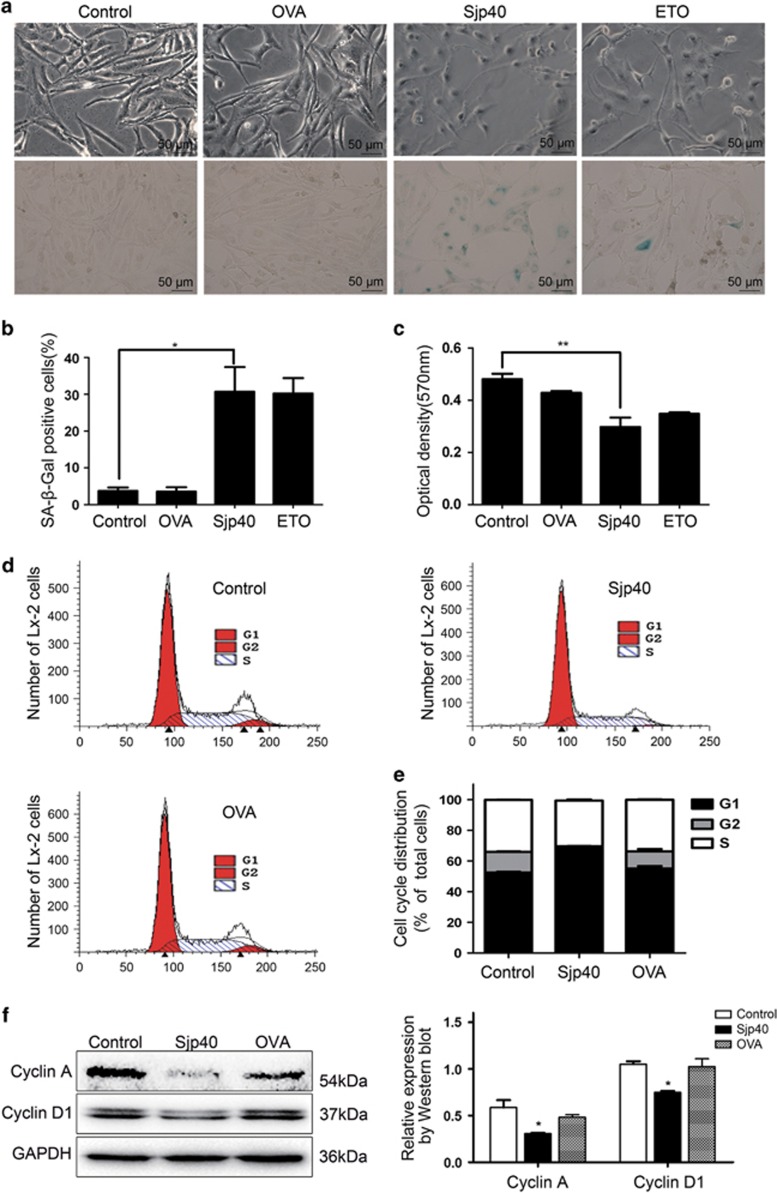
Sjp40 induces a senescence-like phenotype and inhibits cell growth in activated HSCs. (**a**) Effects of Sjp40 (20 *μ*g/ml) on cell senescence in LX-2 cells were determined via senescence-associated *β*-galactosidase assay (original magnification × 200). Bar: 50 *μ*m. (**b**) The graph represents quantitative analysis of the percentage of SA-*β*-Gal-positive cells (%). Data were expressed as the mean±S.E.M. of three or four independent trials. *P<0.05 compared with the control group. (**c**) Effect of Sjp40 (20 *μ*g/ml) on the proliferation of LX-2 cells. LX-2 cells proliferation was examined using the MTT assay. Data were expressed as the mean±S.E.M. of three or four independent trials. **P<0.01 compared with the control group. (**d**) Flow cytometric analysis was applied to analyze Sjp40-induced cell cycle arrest of LX-2 cells in the G_1_ phase. (**e**) Quantitative analysis of cell cycle distribution graph describing the role of Sjp40 in regulating the G_1_ phase of cell cycle arrest. Data were expressed as the mean±S.E.M. of three or four independent trials. (**f**) The expression of Cyclin A and D1 by analysis of western blot. All values were expressed as the mean±S.E.M. of three or four independent trials. *P<0.05 compared with the control group

In our experiments we observed obvious morphological changes in Sjp40-treated LX-2 cells. The cells appeared flat with pyknosis of the nucleus. These morphological features are regarded as common morphologic characteristics of senescent cells (magnification, × 200; Figure 2a). We speculated that Sjp40-induced HSC deactivation may be due to an induction of cellular senescence. We tested this hypothesis by examining effects of Sjp40 on the aging of LX-2 cells using an SA-*β*-Gal assay. Our experiments demonstrated that cells exposed to Sjp40 exhibited dramatically increased SA-*β*-Gal activity (Figures 2a and b). We employed etoposide (ETO), which has been reported to induce the senescence in activated HSCs,^21^ as a positive treatment control. Ovalbumin (OVA) was used as a negative treatment control. Next, we used 3-(4,5-dimethyl-2-thiazolyl)-2,5-diphenyl-2-H-tetrazolium bromide (MTT) assay to determine whether or not Sjp40 has an inhibitory effect on LX-2 cells proliferation. Sjp40 treatment for 96 h markedly suppressed the proliferation capacity of LX-2 cells (Figure 2c). Senescent cells are characterized by cell cycle arrest primarily in the G_1_ phase and occasionally at the S or G_2_ phase.^22^ We used flow cytometry to investigate the effect of Sjp40 on the cell cycle of LX-2 cells. LX-2 cells were preliminarily serum-starved for 24 h in Dulbecco's modified Eagle's medium (DMEM) before Sjp40 treatment and synchronized in the G1 phase (Supplementary Figure S2). Flow cytometry results demonstrated that the number of G_1_-arrested LX-2 cells in the Sjp40-treated group exhibited an obvious increase compared with the control group (Figures 2d and e). Additionally, Sjp40 markedly reduced protein levels of Cyclin A and Cyclin D1 compared with the OVA group. These results demonstrate that Sjp40 is indeed arresting the LX-2 cells in the G1 phase. Together, these experiments confirmed that the treatment with Sjp40 resulted in cellular senescence in HSCs via inhibiting cell growth and promoting cell cycle arrest.

### Sjp40 induces HSC senescence via a p53/p21-dependent mechanism

**Figure 3:**
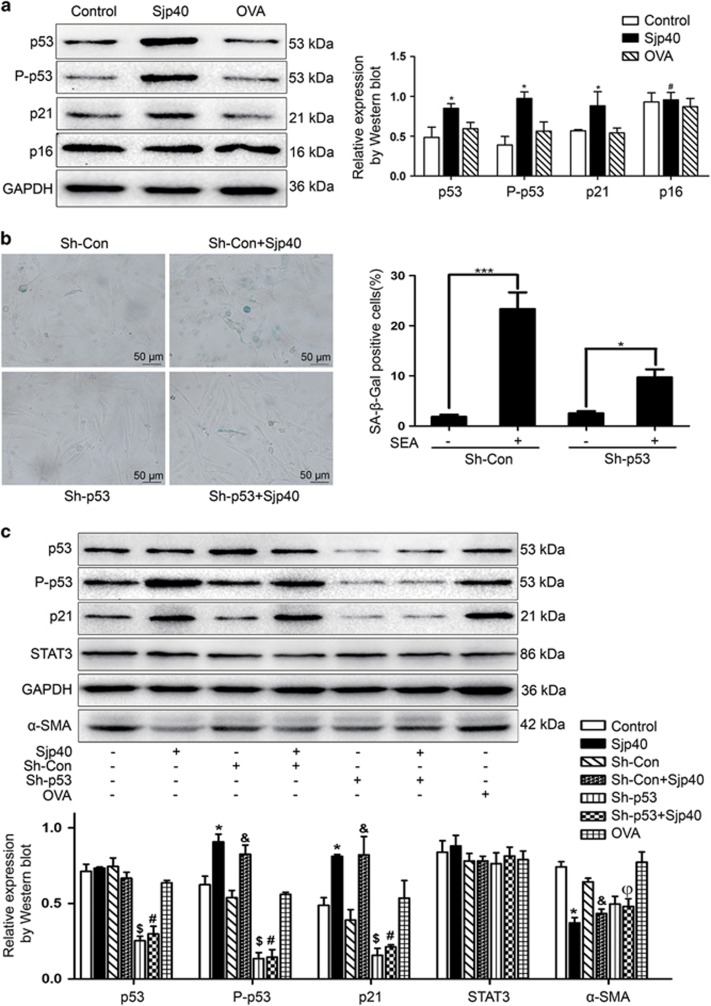
Sjp40-induced senescence is dependent on the p53 and p21 pathway. (**a**) Western blot analysis of the expression of p53, P-p53, p21 and p16 in LX-2 cells treated with Sjp40. Data were expressed as the mean±S.E.M. of three or four independent trials. *P<0.05 compared with the control group. ^#^*P*>0.05 compared with the control group. (**b**) Knockdown of p53 rescued the Sjp40-induced senescence analyzed by SA-*β*-Gal assay, and quantitative analysis of the percentage of SA-*β*-Gal-positive cells (%) was expressed as the mean±S.E.M. of three or four independent trials. *P<0.05 compared with ShRNA-p53 (Sh-p53). ***P<0.001 compared with ShRNA-Control (Sh-Con). Bar: 50  *μ*m. (**c**) LX-2 cells were transfected with Sh-p53 or Sh-Con and additionally treated with or without Sjp40 for 48 h. The protein expression was investigated by western blot assay and data were expressed as the mean±S.E.M. of three or four independent trials. *P<0.05 compared with the control group; ^&^*P*<0.05 compared with the Sh-con group. ^$^*P*<0.05 compared with the control group. ^#^*P*>0.05 compared with the Sh-p53 group. ^ϕ^*P*<0.05 compared with the Sjp40 group

Cellular senescence is mediated by the p53/p21- and p16-dependent pathway.^23^ These pathways are present in the majority of senescent cells and are viewed as both tumor suppressors and biomarkers of aging.^24^ To elucidate the underlying molecular mechanism of Sjp40-induced senescence in HSCs we conducted experiments to examine markers of senescence. Total proteins were extracted from LX-2 cells treated with or without Sjp40 and then assayed by western blot using antibodies against p53, P-p53, p21 and p16. Sjp40 largely enhanced the expression of P-p53 and p21 but did not appear to affect the expression level of p16. To confirm the influence of Sjp40 on the expression of P-p53, p53 was knocked down using an ShRNA specific to p53. Once again we used SA-*β*-Gal staining to measure Sjp40-induced senescence in LX-2 cells. We found that cellular senescence was markedly attenuated following p53 knockdown. Consistent with the finding, western blot analysis showed that p53 silencing markedly reduced the expression of P-p53 and p21 (Figure 3). These results indicated that Sjp40 induced senescence in LX-2 cells via the p53/p21 signaling pathway.

### The Sjp40-mediated HSC senescence is linked with STAT3

**Figure 4:**
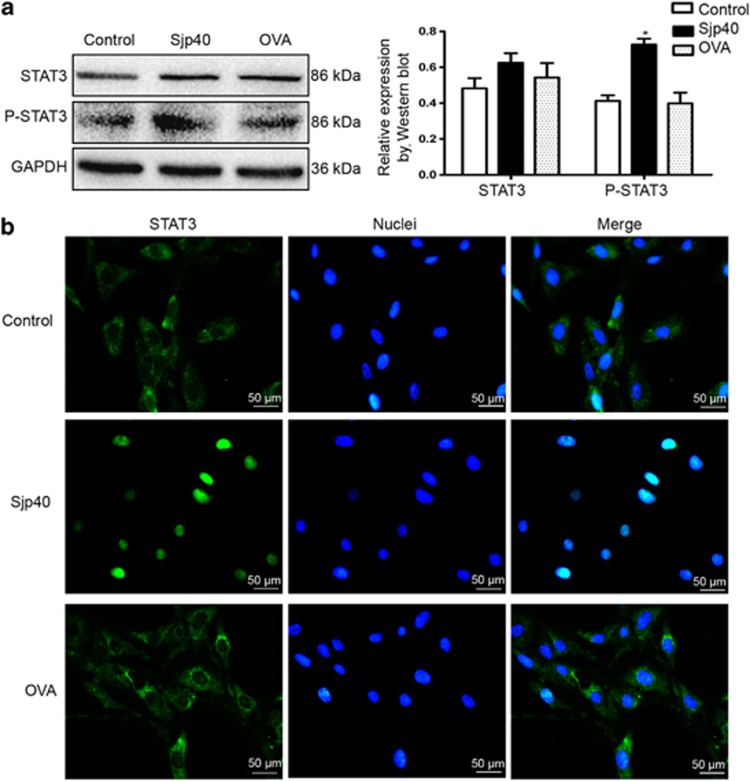
STAT3 is required for Sjp40-induced senescence in LX-2 cells. (**a**) Western blot analysis of the expression of STAT3 and P-STAT3 in LX-2 cells treated with the Sjp40. Data were expressed as the mean±S.E.M. of three or four independent trials. *P<0.05 compared with the control group. (**b**) The Sjp40-induced translocation of STAT3 was detected by immunofluorescence assay. The cells were photographed using a fluorescence microscope. Bar: 50 *μ*m

**Figure 5:**
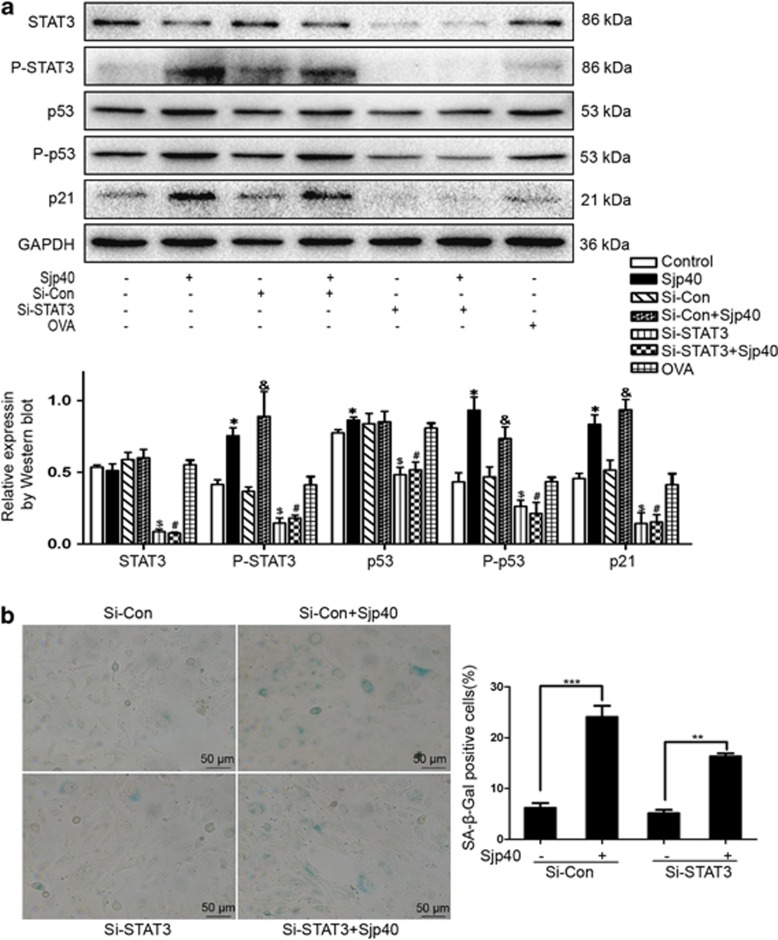
STAT3 is involved in Sjp40-induced HSC senescence and regulating the senescence-associated signaling pathway. (**a**) LX-2 cells were transfected with Si-STAT3 (Si-STAT3) or Si-Control (Si-Con) and additionally treated with or without Sjp40. The protein expression was investigated by western blot assay and data were expressed as the mean±S.E.M. of three or four independent trials. *P<0.05 compared with the control group. ^#^*P*>0.05 compared with the Si-STAT3 group. ^$^*P*< 0.05 compared with the Si-Con group. ^&^*P*<0.05 compared with the Si-Con group. (**b**) Knockdown of STAT3 rescued the Sjp40-induced senescence analyzed by SA-*β*-Gal assay and quantitative analysis of the percentage of SA-*β*-Gal-positive cells (%) was expressed as the mean±S.E.M. of three or four independent trials. **P<0.01 compared with the Si-STAT3 group. ***P<0.001 compared with the control group. Bar: 50 *μ*m

STAT3 is a signaling component that is upstream of p53. STAT3 has been reported to promote cellular senescence by inducing an upregulation of p53 and p21.^25^ Considering STAT3's role as a regulator of cellular senescence, we tested whether or not STAT3 was involved in the activation of p53 signaling triggered by Sjp40. We used western blot analysis to examine protein levels of STAT3 and P-STAT3. Sjp40 markedly increased the expression of P-STAT3 compared with the untreated group (Figure 4a). Moreover, in the presence of Sjp40 we observed an obvious translocation of STAT3 from the cytoplasm to the cellular nucleus. Conversely, in the absence of Sjp40 the majority of STAT3 was primarily located in the cytoplasm (Figure 4b). Next, we analyzed the expression of P-STAT3, p53, P-p53 and p21 following STAT3 knockdown. As illustrated in Figure 5a, the increased protein expression of P-STAT3, P-p53 and p21 that was triggered by Sjp40 treatment could be reversed by Si-STAT3 but not by Si-Con. To confirm the role of STAT3 in Sjp40-induced senescence we analyzed cell growth in LX-2 cells where STAT3 was knocked down using a SA-*β*-Gal assay. Si-RNA knockdown of STAT3 in cells treated with Sjp40 reduced the number of SA-*β*-Gal-positive cells and partially rescued the Sjp40-induced senescence in LX-2 cells. Knockdown of STAT3 did not appear to affect TLR-4 expression (Supplementary Figure S3). These experiments confirmed the critical role of STAT3 in regulating the cellular aging induced by Sjp40. Together, these experiments indicate that STAT3 mediated Sjp40-induced senescence in HSCs via p53/p21 signaling.

### TLR-4 contributes to cellular senescence induced by Sjp40 in LX-2

**Figure 6:**
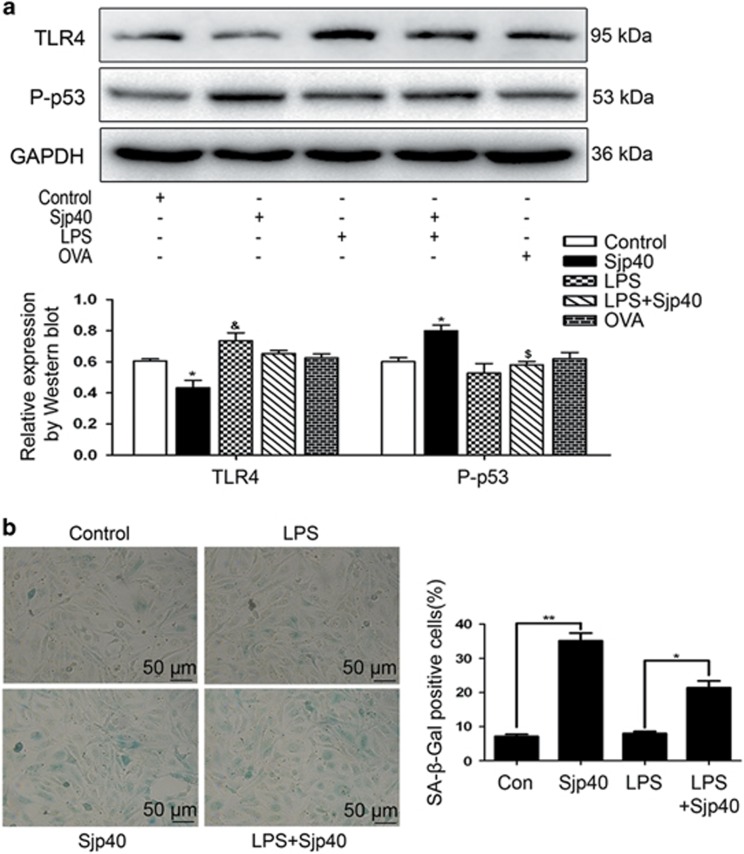
The downregulation of TLR-4 in Sjp40-treated LX-2 is associated with cellular senescence. (**a**) Western blot analysis of the expression levels of P-p53 and TLR-4 in LX-2 cells treated with the Sjp40. Data were expressed as the mean±S.E.M. of three or four independent trials. *P<0.05 compared with the control group; ^&^*P*<0.05 compared with the control group; ^$^*P*<0.05 compared with the Sjp40-treated group. (**b**) Enhanced expression of TLR-4 by LPS restored the Sjp40-induced senescence analyzed by senescence-associated *β*-galactosidase assay (original magnification × 200) and the graph also reveals quantitative analysis of the percentage of SA-*β*-Gal-positive cells (%) expressed as the mean±S.E.M. of three or four independent trials. *P<0.05 compared with the LPS-treated group; **P<0.01 compared with control group. Bar: 50 *μ*m

TLR-4, as a receptor for LPS, has been implicated in the regulations of HSC activation and collagen type I production during liver fibrosis.^26^ LPS mostly induced the expression of TLR-4 at a concentration of 0.1 *μ*g/ml for 24 h (Supplementary Figure S4). In the current study, some data demonstrated that Sjp40 suppressed HSC activation via the inhibition of collagen type I and *α*-SMA. In addition, we also found that the treatment of Sjp40 largely inhibited the expression of TLR-4 (Figure 6a). Thus, we speculated that TLR-4 elicits a critical effect in regulating LX-2 cells senescence triggered by Sjp40. To test this hypothesis, we used LPS to stimulate the expression of TLR-4. Protein levels of P-p53, the most critical aging-associated molecule, were significantly inhibited by LPS stimulation. SA-*β*-Gal assay also showed that LPS stimulation reduced the number of SA-*β*-Gal-positive cells and partially rescued the Sjp40-induced senescence in LX-2 cells. These experiments demonstrated that Sjp40-induced senescence is mediated via TLR-4 in LX-2 cells.

### Sjp40-induced senescence in LX-2 cells is easily targeted by NK cells

**Figure 7:**
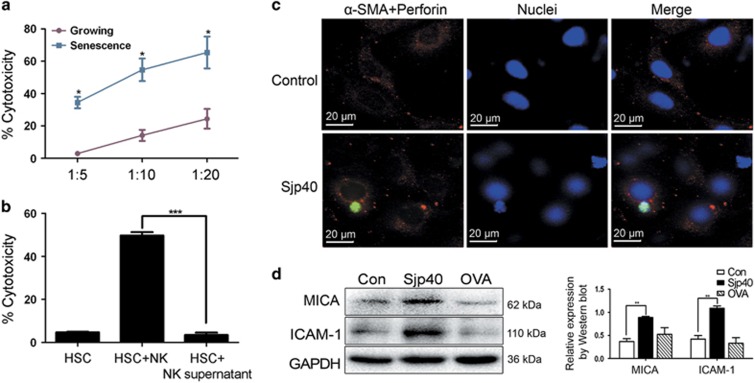
Sjp40-induced senescent LX-2 cells were easily targeted by NK cells. (**a**) NK cells cytotoxicity measured by crystal violet staining of remaining adherent LX-2 cells was based on crystal violet quantification at OD595. Data were expressed as the mean±S.E.M. of three or four independent trials. *P<0.05 compared with the control group. (**b**) Senescent LX-2 cells were co-cultured with YT cells or the supernatants from YT cells medium for 12 h. Then, induction of NK cytotoxicity was measured by crystal violet staining of remaining adherent cells. ***P<0.001 compared with the NK supernatant group. (**c**) The specific NK-HSC adhesion was observed in growing or senescent group by immunofluorescence assay. The cells were photographed using a fluorescence microscope. In these representative images, the *α*-SMA appears red and the perforin appears green. (**d**) Western blot analysis of the expression levels of ICAM-1 and MICA in LX-2 cells treated with the Sjp40. Data were expressed as the mean±S.E.M. of three or four independent trials. **P<0.05 compared with the control group

In the progression of liver fibrosis NK cells have specific protective effects that selectively eliminate activated HSCs. This effect is particularly apparent in senescent-activated HSCs or early activated HSCs. We hypothesized that when HSCs age the expression of intercellular adhesion molecule (ICAM-1) and ligand molecule of the NK cell receptor might increase. To test this hypothesis we analyzed adhesion molecule ICAM-1 and ligand molecule MICA protein levels in LX-2 cells exposed to Sjp40 by western blot. Following Sjp40 stimulation the protein expression of ICAM-1 and MICA in LX-2 cells significantly increased (Figure 7d). To observe the elimination activity of NK cells against senescent-activated HSCs YT cells (a human NK cell line) were used to co-culture with growing or Sjp40-induced senescent LX-2 cells at different T : E ratios. LX-2 cells that were induced to senescence by Sjp40 were much more sensitive to NK-mediated clearance (in a dose-dependent manner) compared with untreated controls (Figure 7a). We then investigated the potential interaction between LX-2 and YT cells by immunofluorescence assay. We observed specific NK-HSCs adhesion after 2 h co-culture in the Sjp40-treated group and very little NK-HSC adhesion in the control group (Figure 7c). In conclusion, Sjp40-induced senescence might enhance the elimination effect of NK against HSCs via cell–cell adhesion and receptor–ligand activation. These experiments indicated that the NK-cell-mediated elimination of senescent HSCs was mediated by cell–cell contact mechanism rather than by secreted factors. To confirm our hypothesis, we co-incubated senescent LX-2 cells with YT cells or the supernatant of YT cells medium for 12 h and then we assessed the NK-mediated cytotoxicity effect via crystal violet staining (Figure 7a) and ELISA 5-bromo-2-deoxyuridine (BrdU) kit (Supplementary Figure S5). We found that YT cells could eliminate the aging LX-2 cells, but supernatants from YT cells had a limited or no impact on cell clearance in senescent LX-2 (Figure 7b).

## Discussion

Liver fibrosis is a common forerunner of many chronic liver diseases, resulting from excessive wound healing and is characterized with ECM components.^23^ Until recently, the primary belief in the literature was that hepatic fibrosis is irreversible, but new evidence has demonstrated that liver fibrosis can be suppressed and potentially even reversed.^26^ Blocking collagen fibril formation^23^ and the prevention of the epithelial–mesenchymal transition (EMT) process^27^ that transforms epithelial cells into myofibroblast (MFB) and the activation of NK cells on HSCs are hallmarks of fibrosis regression.^6^ Together these processes will eventually alleviate hepatic fibrosis, but the suppression of HSC activity and reduction of the number of activated HSCs are a great importance. Activated HSCs are the main effector cell in the progression of liver fibrosis. They are a major source of ECM and they also secrete large amounts of pro-inflammatory and pro-fibrogenic cytokines.^1^ Thus the inhibition and removal of activated HSCs are critical strategies for liver fibrosis treatment. This may be employed by the induction of cellular senescence,^7^ encouraging cell apoptosis,^5^ and enhancing the clearance activity of immunocytes on HSCs.^28^

Cellular senescence refers to an irreversible cell cycle arrest state that can limit cell proliferation and growth.^24^ It is widely accepted that cell senescence could serve as an effective strategy for tumor suppression.^29^ Recent research has promoted the idea that induction of cellular senescence could also play a vital suppressive role in other non-neoplastic diseases such as liver fibrosis. Additional studies have found that the induction of aging in activated HSCs might limit hepatic fibrosis,^25^ whereas the restriction of the senescence program could aggravate liver fibrogenesis.^7^ Once HSCs undergo an EMT and become MFB, these cells possess potent proliferation activity and produce large amounts of ECM which promotes fibrosis formation. Induction of senescence could restrain activated-HSC growth and reduce the cellular population, which may result in the reduction of ECM secretion and regression of liver fibrosis.^30^ In addition, the secretory characteristics change in senescent activated-HSCs. Fibrosis-promoting components such as collagen types I and IV are suppressed while components that inhibit hepatic fibrosis are increased. Matrix metalloproteinases (MMP), which effectively degrade ECM components such as MMP-1 and MMP-3, undergo enhanced expression in senescent activated-HSCs.^7^ Senescent activated-HSCs also produce more immunoregulatory molecules that could activate NK cell function and enhance the killing activity of NK cells against HSCs and contribute to the alleviation of fibrosis.^23^

The p53/p21/CDK2/P-Rb, Ras/p16/CDK4/P-Rb and Skp2/p27/P-Rb pathways regulate cellular senescence.^23^ Historically, the p53/p21/CDK2/P-Rb pathway has been recognized as one of the most important signal transduction pathways for regulating and maintaining cell growth arrest.^31^ The p53 and p21 are the vital effectors in the p53/p21/CDK2/P-Rb signaling pathway. This pathway can be activated by glucocorticoids to induce the senescence of primary human tenocytes.^23^ p53 is one of the most important tumor suppressor genes in cancer-associated diseases and plays a crucial role in regulating cellular senescence.^32^ p53 activation signals a DNA damage response, which is a common step in the process of senescence arrest.^23^ Following the downstream target of p53, p21 is activated and triggers the cell cycle arrest.^22^ In this way, p21 is viewed as a critical mediator in DNA damage-induced senescence and p53 activation.^33^ Studies have revealed that p53/p21 signaling is also associated with liver fibrosis. The activation of the p53/p21 signaling pathway could induce HSC senescence, which could result in the inhibition of HSC proliferation and a reduction in the expression of collagen genes.^23^ Work by our laboratory is consistent with these data and we demonstrated that p53 and p21 are involved in the HSC senescence triggered by Sjp40. Of note, the silencing of p53 could reverse the Sjp40-induced senescence in LX-2 cells.

Signal transducer and activator of transcription 3 (STAT3) plays a variety of roles in regulating cellular function.^34^ In some conditions, STAT3 is involved in cell survival and growth via activating pro-proliferative cytokines.^35^ Under other conditions, STAT3 is a key regulator in cellular senescence and growth arrest.^36, 37^ As Kojima *et al.*^38^ reported, STAT3 is required for IL-6/gp 130-induced senescence in human lung fibroblasts. In the hepatic fibrosis mouse model the role of STAT3 on senescence in activated HSCs has studied. Kong *et al.*^25^ performed experiments that showed that IL-22 induced activated-HSC senescence in STAT3-dependent mechanism through the upregulation of p53 and p21. Consistent with the literature we found that Sjp40 could induce the activation of STAT3 and Si-RNA knockdown of STAT3 could partially rescue the Sjp40-induced senescence in LX-2 cells, suggesting the vital role of STAT3 in cellular senescence.

The LPS/TLR-4 signaling pathway is closely related to HSC activation during hepatic fibrosis or liver injury.^39^ Recently, research has found that TLR-4-dependent signaling pathways are linked with antifibrotic effect. In this work HSCs activation-associated makers could be decreased via the reduction of TLR-4 protein level. As Liu *et al.*^40^ demonstrated, plumbagin could effectively regress hepatic fibrosis by downregulating the NF-*κ*B/TLR-4 pathway in CCl4-induced rats models. Liu *et al.*^41^ reported that the antifibrosis capability of dioscin was due to a reduction in TLR-4 expression that resulted in decreased levels of *α*-SMA, collagen type I, TGF-*β*1 and fibronectin. In line with those studies, our data also indicated that Sjp40 could inhibit HSC activation through the reduction of *α*-SMA and collagen type I expression. Notably, HSC inactivation was accompanied by decreased expression of TLR-4. Furthermore, Fu-ping Wang *et al.*^42^ found that activation of the TLR-4-dependent signal pathways, JNK and PI3K/AKT, facilitated HSC proliferation. In the current study we tested whether the attenuated proliferation in HSCs triggered with Sjp40 resulted from decreased expression of TLR-4. We found that LPS partially rescued the Sjp40-induced senescence through increasing the level of TLR-4. Together our results suggest a model in which TLR-4 contributes to cellular senescence via regulating P-p53 expression.

In the liver microenvironment, many kinds of immune cells, including Kupffer cells, T lymphocytes and NK cells participate in reversing liver fibrosis.^43^ Melhem *et al.*^44^ discovered that NK cells have an antifibrotic activity via stimulation of HSC clearance in CCl_4_-induced mice models and *in vitro* human NK/HSC co-cultures. It has been also shown that NK cells could directly kill activated HSCs via an NKG2D-mediated mechanism in mouse models. Jeong *et al.*^45^ found that the cytotoxicity of NK against HSCs was decreased in STAT1^−/−^ mice, which was likely due to the downregulation of NKG2D expression on STAT1^−/−^ NK cells. The upregulation of NKG2D expression in NK cells exposed to polyinosinic acid (Poly (I:C)) or IFN-*γ* may promote the combination of NKG2D and its ligands in HSCs, which enhance the cytotoxicity of NK cells against activated HSCs.^46^ In this scenario NK cells could selectively eliminate activated HSCs, particularly senescent activated HSCs. Once HSCs age the expression of cell-surface adhesion molecules and ligand molecules might increase. Krizhanovsky *et al.*^7^ suggested that the increased expression of adhesion molecules and NK cell receptor ligands in senescent activated HSCs contributes to the cell–cell adhesion and NK cell activation. In our studies, we used a human NK cells line (YT cells) and co-cultured them with growing LX-2 cells or Sjp40-induced senescent LX-2 cells in order to observe the elimination activity of NK cells. Our results showed that Sjp40-induced senescent LX-2 cells were targeted by YT cells. Additional experiments conclusively indicated that this is likely due to the high expression of adhesion molecule ICAM-1 and NKG2D ligand molecule MICA in senescent LX-2 cells. These conditions might improve the cytotoxicity of NK against HSCs via cell–cell adhesion and the receptor–ligand pathway.

In summary, our current findings clearly demonstrated that Sjp40 could markedly ameliorate LX-2 cells activation and trigger cell senescence. We also showed that Sjp40-induced senescence was mediated by the STAT3/p53/p21 signaling pathway in LX-2 cells and senescent cells are more sensitive to YT cells. Together our data have provided novel insights into the mechanisms of antifibrosis.

## Materials and Methods

### Production and purification of Sjp40

The open reading frame of the Sjp40 gene from *S. japonicum* eggs was cloned into a pET-28a (+) vector and transformed into *Escherichia coli* BL21 (DE3). Then the recombinant Sjp40 protein was expressed and purified by the Ni-NTA His•Bind Resin (Novagen, Merck, Darmstadt, Germany) according to the instructions. After identified by western blot, the endotoxin of Sjp40 recombinant protein was removed using polymyxin B-agarose beads following our previous protocol.^20^ Sjp40 was dissolved in PBS.

### Isolation and culture of HSCs

Primary HSCs were isolated from the livers of normal mice according to our previous study.^47^ Primary HSCs were activated by TGF*β*1 (5 ng/ml) and *in vitro*. The activated cells were treated with Sjp40 (20 *μ*g/ml) for 48 h. The human hepatic stellate cell line (LX-2) was obtained from Xiang Ya Central Experiment Laboratory (Changsha, Hunan, China) and maintained in Dulbecco's modified Eagle's medium DMEM (Gibco, Thermo Fisher Scientific, Waltham, MA, USA) with 10% fetal bovine serum (Invitrogen, Thermo Fisher Scientific, Waltham, MA, USA). Cells were cultured in a humidified incubator at 37 °C with 5% CO_2_. Cells were stimulated with the addition Sjp40 (20 *μ*g/ml) or LPS (0.1 *μ*g/ml) in complete media or media only control.

### Quantitative real-time PCR (qRT-PCR)

Total RNA was extracted and transcribed into cDNA as previously described.^20, 47^ cDNA products worked as the template for qRT-PCR analysis with a SYBR Premix Ex Taq Kit (TAKARA, Dalian, China) on the Eco Real-Time PCR Sequence Detection System (Illumina, San Diego, CA, USA). All samples were run in triplicate and the relative expression levels were determined by normalization to b-actin and presented as fold increase or decrease relative to the controls. *α*-SMA, forward: 5′-CACTGCCTTGGTGTGTGACAAT-3′ reverse: 5′-CGTAGCTGTCTTTTTGTCCCATTC-3′. Collagen type I, forward: 5′-CAAGGTGTTGTGCGATGACG-3′ reverse: 5′-TGGTTTCTTGGTCGGTGGGTG-3′.

### MTT assay

LX-2 cell proliferation was evaluated by MTT (Sigma, St. Louis, MO, USA) assay. Cells (1 × 10^4^ cells/well) were seeded in 96-well plates for 24 h. Each well was filled with the appropriate stimulus and cells were cultured for 72 h at 37 °C. MTT (5 mg/ml) was added and incubated for 4 h. The absorbance at 570 nm was measured using an ELISA reader (Bio-Tek, Vermont, USA).

### Western blot

Total proteins were extracted from LX-2 cells using standard methods. Protein concentration was quantified by Bradford method (Sangon, Shanghai, China). Protein samples were separated by SDS-PAGE (8–12%), transferred onto Shanghai, PVDF membranes (Merck, Darmstadt, Germany) and blocked with 5% non-fat dry milk. Membranes were incubated with specific primary antibody at 4 °C overnight and then incubated with an appropriate second antibody at room temperature. A chemiluminescence (ECL) kit (Merck, Darmstadt, Germany) was used to detect target proteins. Protein bands were normalized to GAPDH and protein expression was quantified by Image J (National Institutes of Health, Bethesda, MD, USA).

### Cytotoxicity assay

Growing or senescent LX-2 cells were plated into 96-well plates for 24 h. Then YT cells were co-cultured with LX-2 cells at different target: effector ratios (T:E) (1:5, 1:10, 1:20) for 12 h. NK cell cytotoxicity measured by crystal violet staining of remaining adherent LX-2 cells was based on crystal violet quantification at OD quantification at OD595. In parallel, cytotoxicity was measured by BrdU incorporation using the Cell Proliferation ELISA, BrdU (colorimetric) kit (Roche Diagnostics, Basel-Stadt, Switzerland) according to the manufacturer's instructions. The absorbance at 450 nm was measured in an ELISA reader.

### Flow cytometric analysis

LX-2 cells were preliminary serum-starved for 24 h in DMEM before Sjp40 treatment and synchronized in the G1 phase. And then the medium was replaced with DMEM containing 10% FCS with or without Sjp40 (20 *μ*g/ml). Following stimulation cells were trypsinized and resuspended in 0.3 ml PBS, fixed with 0.7 ml anhydrous ethanol at 4 °C overnight. Fixed cells were resuspended in 0.9 ml PBS and incubated with Rnase A (50 *μ*g/ml; TAKARA, Dalian, China) for 20 min at 37 °C. Cellular DNA was labeled with propidium iodide (50 *μ*g/ml; Biosharp, Hefei, Anhui, China) for 30 min at 4 °C. Samples were filtered to remove cell clumps and analyzed by flow cytometry using a FACSCalibur instrument (BD Biosciences, San Jose, CA, USA).

### RNA interference

Cells were seeded into six-well plates for 24 h. To knock down p53 expression, ShRNA-p53 (Sh-p53; Genechem, Shanghai, China) or ShRNA-control (Sh-con; Genechem) were transfected into LX-2 cells. Cells were treated with or without Sjp40 (20 *μ*g/ml) 24 h after transfection. Human small-interfering RNAs against STAT3 (Si-STAT3) and a scrambled sequence control (Si-con) were purchased from GenePharma (Shanghai, China). LX-2 cells were transfected with Si-STAT3 or Si-con for 6 h using Lipofectamine 2000 (Invitrogen) and then were treated with or without stimulus for 48 h.

### SA-*β*-Gal assay

LX-2 cells were plated into six-well plates for 24 h and then stimulated. Cells were stained using a senescence-associated *β*-galactosidase staining kit (Genmed, Shanghai, China) according to the manufacturer's instructions.

### Immunofluorescence assay

LX-2 cells were seeded in six-well plates overnight before stimulus. Cells were fixed with 4% paraformaldehyde for 1 h and permeabilized with 0.1% Triton-100 for 5 min. After blocking in 5% BSA for 2 h at room temperature the cells were incubated with anti-STAT3 antibody (1 : 50) at 4 °C overnight. A fluorescently-labeled second antibody was incubated with cells for 1.5 h at 37 °C. The cellular nuclei also stained with Hoechst 33342 for 20 min and cells were photographed using a fluorescence microscope.

### Statistical analysis

Data analysis was performed using one-way ANOVA or the independent samples *T*-test. All data were expressed as the mean±S.E.M. of three or four independent trials to determine the significant differences. *P*<0.05 was considered statistically significant.

## Abbreviations

HSCs: hepatic stellate cells
Sjp40: egg antigen p40 of *Schistosoma japonicum*
*α*-SMA: *α*-smooth muscle actin
ECM: extracellular matrix
SA-*β*-Gal staining: senescence-associated *β*-galactosidase staining
STAT3: signal transducer and activator of transcription 3
TLR-4: Toll-like receptor 4
