# Supplementary material for: Egg antigen p40 of Schistosoma japonicum promotes senescence in activated hepatic stellate cells by activation of the STAT3/p53/p21 pathway
Source: Cell Death Dis. 2016 Jul 28;7(7):e2315–. doi: 10.1038/cddis.2016.228 (PMC4973363; doi:10.1038/cddis.2016.228)
Supplement: Supplementary Figures [file cddis2016228x1.doc]

**Figure legends of supplemental figures**

**Figure S1 Expression and purification of recombinant Sjp40 protein. (a)** SDS-polyacrylamide gel of recombinant pET-28a-Sjp40 with a His ladder after [purification](javascript:void(0);) by Ni-NTA column M: Protein Marker; 1: Flowthrough after passing the supernatant through an Ni-NTA column; 2.Wash; 3-7: 50, 100, 250, 500, and 750mM Imidazole elution fraction. **(b)** Western blot analysis using mouse anti-His monoclonal antibody as primary Ab.

**Figure S2 Synchronization of cells.** LX-2 cells were preliminary serum-starved for 24 h in DMEM before Sjp40 treatment to synchronize in G1 phase by Flow cytometric analysis.

**Figure S3 The role of STAT3 on the expression of TLR4.** LX-2 cells were transfected with Si-STAT3 (Si-STAT3) or Si-Control (Si-Con) and additionally treated with or without Sjp40. The protein expression was investigated by Western blot assay and date was expressed as the mean ± SEM of three or four independent trials. #*P*>0.05 compared to Si-Con group.

**Figure S4 LPS induced the expression of TLR4.** **(a)** LX-2 cells were exposed at the several LPS dosages (0.01, 0.1 and 1 μg/ml). **(b)** LX-2 cells were exposed to LPS at several time periods (6h, 12h, 24h).

**Figure S5 Sjp40-induced senescent LX-2 cells were easily targeted by NK cells.** Senescent LX-2 cells were co-cultured with YT cells or the supernatants from YT cells medium for 12 h. Then, induction of NK cytotoxicity was measured by ELISA 5-bromo-2-deoxyuridine (BrdU) kit.
