# Supplementary figures and images for: Egg antigen p40 of Schistosoma japonicum promotes senescence in activated hepatic stellate cells by activation of the STAT3/p53/p21 pathway
Source: Cell Death Dis. 2016 Jul 28;7(7):e2315–. doi: 10.1038/cddis.2016.228 (PMC4973363; doi:10.1038/cddis.2016.228)

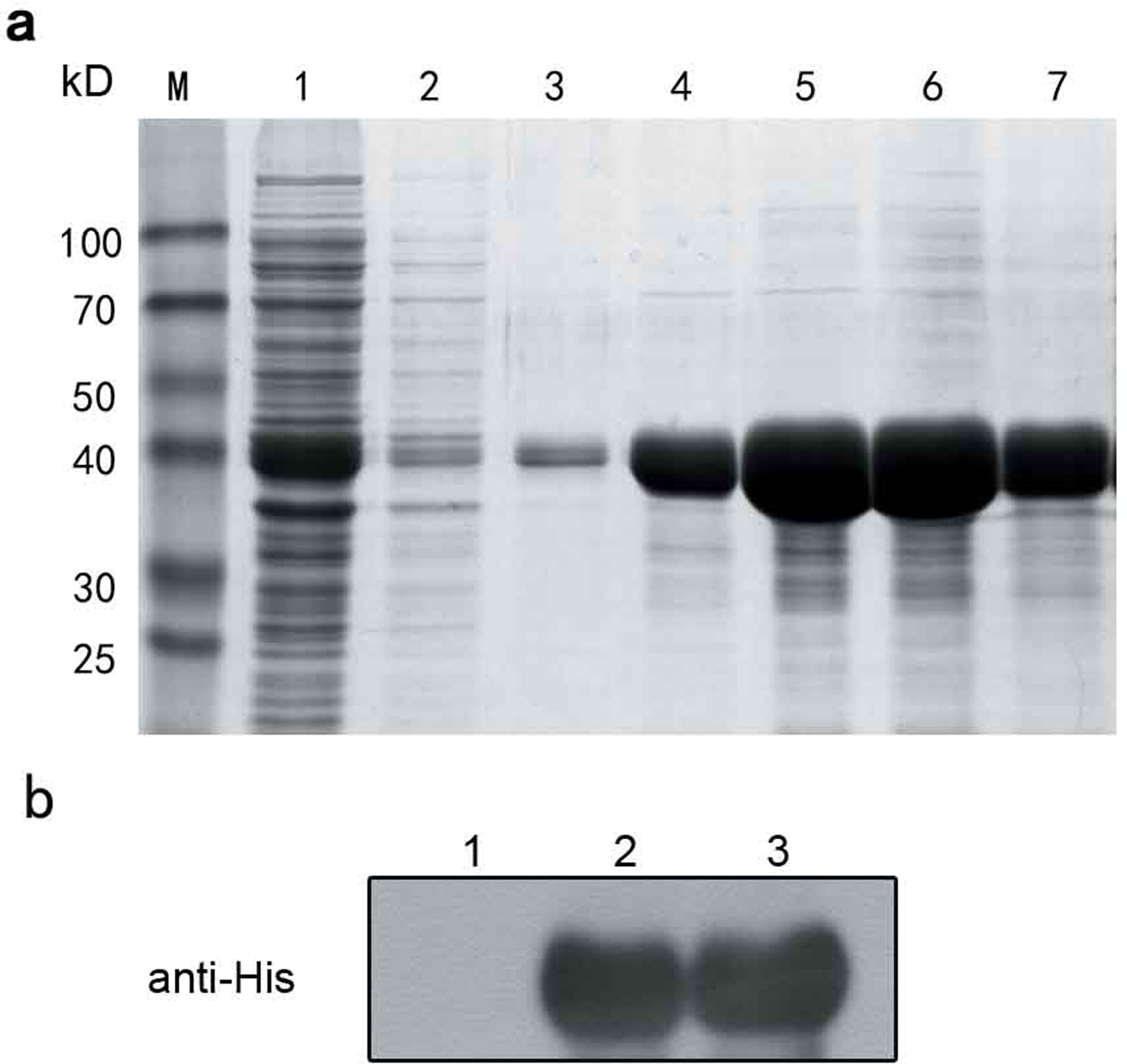

Supplement: Supplementary Figure S1 [file cddis2016228x2.tif]

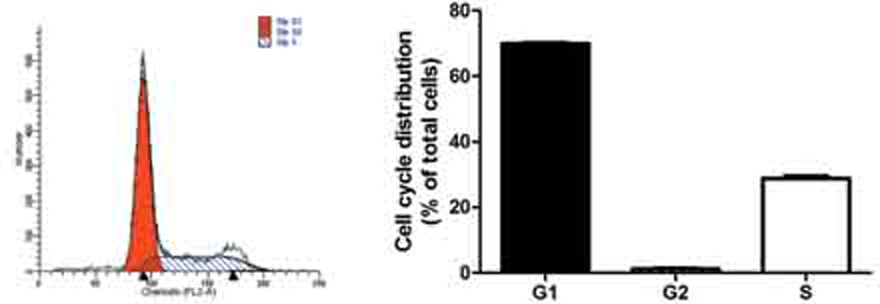

Supplement: Supplementary Figure S2 [file cddis2016228x3.tif]

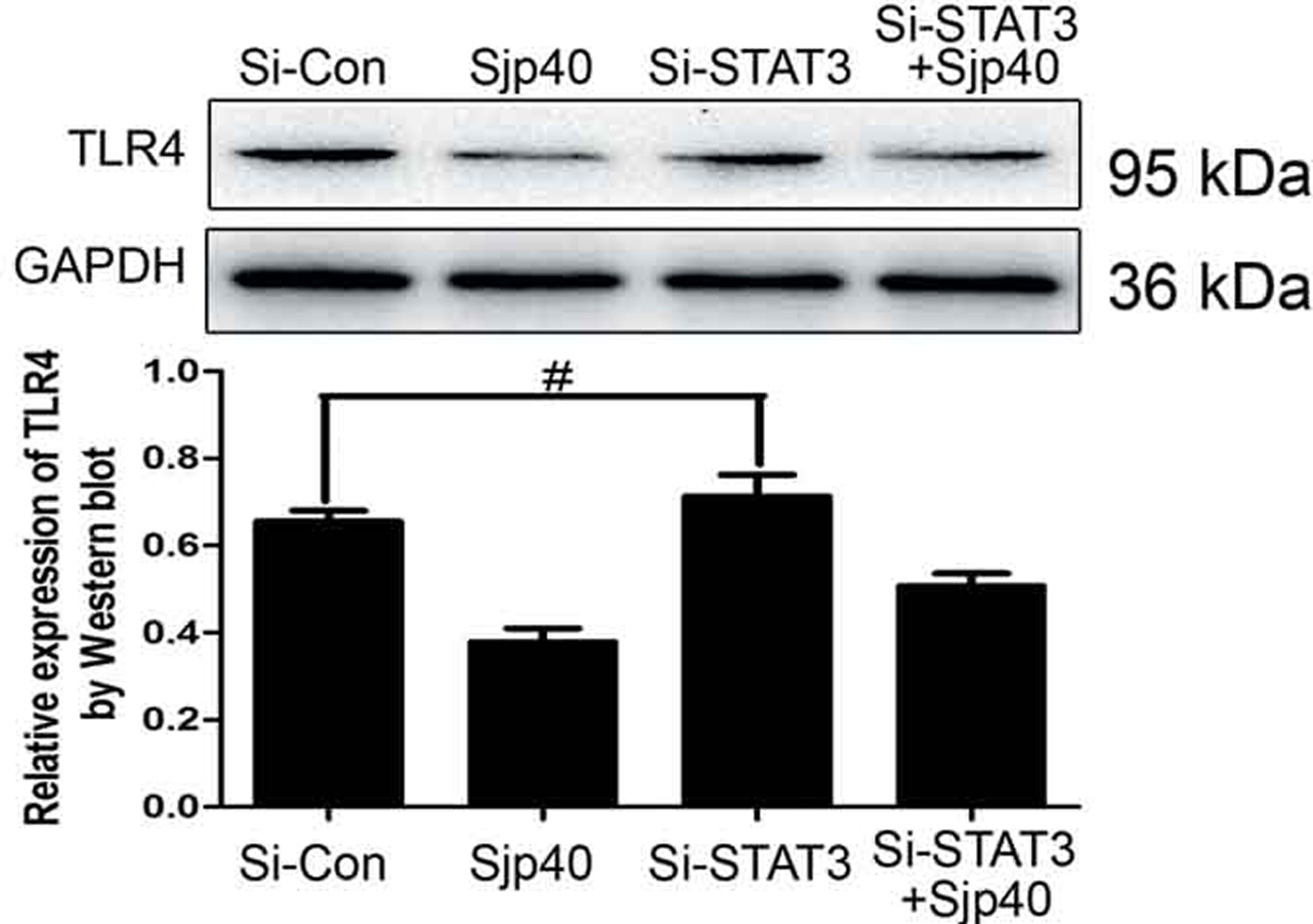

Supplement: Supplementary Figure S3 [file cddis2016228x4.tif]

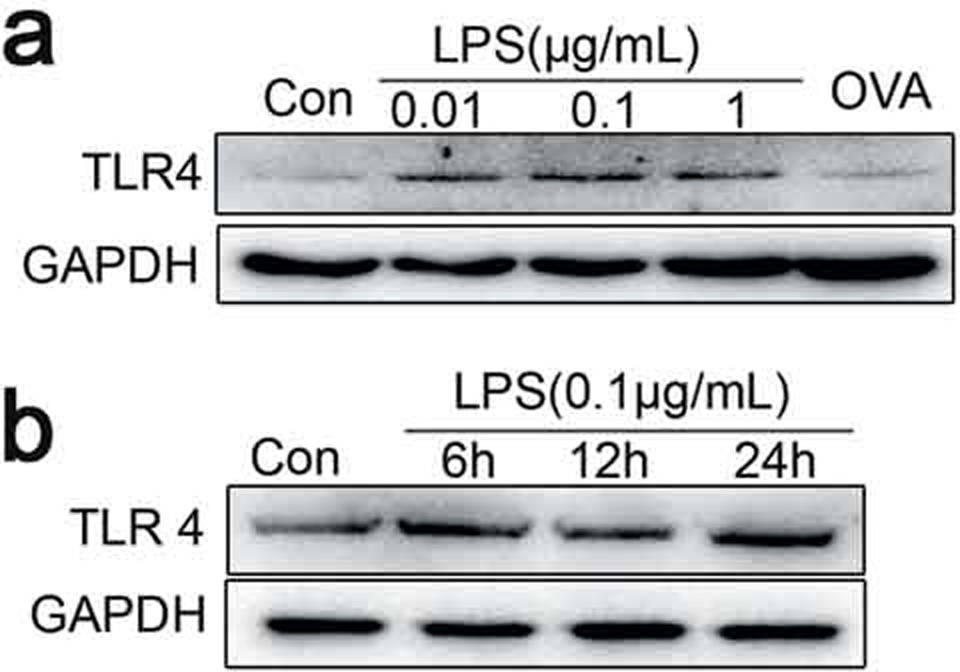

Supplement: Supplementary Figure S4 [file cddis2016228x5.tif]

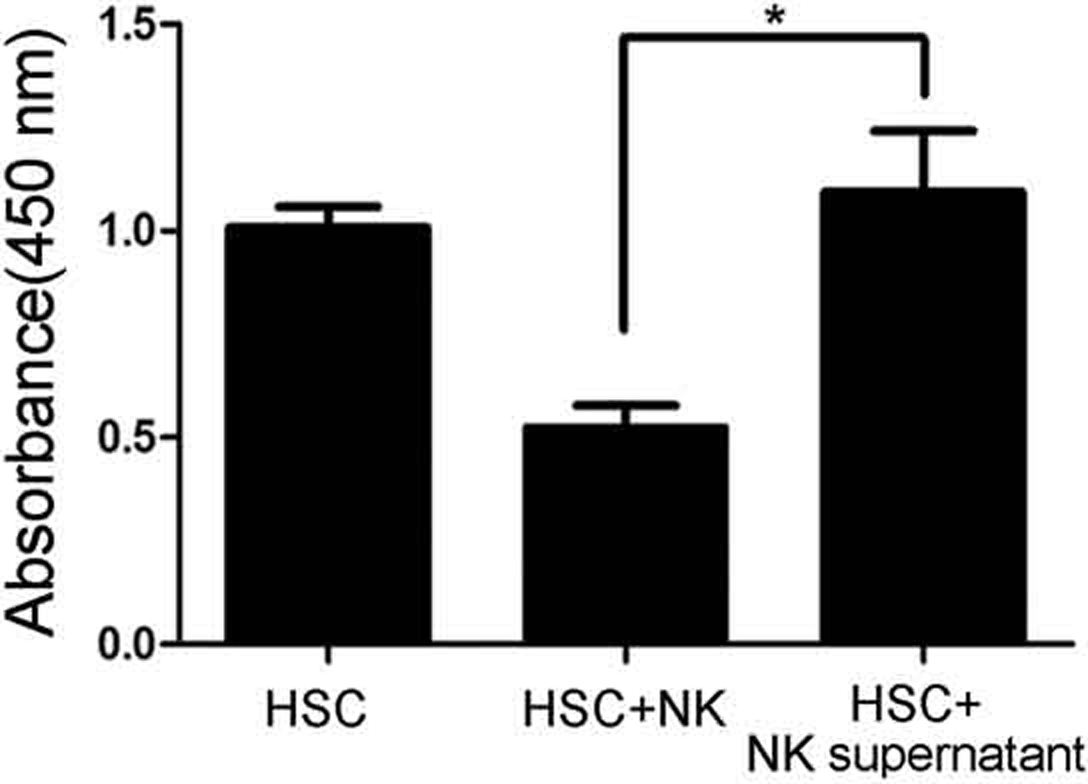

Supplement: Supplementary Figure S5 [file cddis2016228x6.tif]
